# A new timepiece: an epigenetic mitotic clock

**DOI:** 10.1186/s13059-016-1085-y

**Published:** 2016-10-19

**Authors:** Brock C. Christensen, Karl T. Kelsey

**Affiliations:** 1 Department of Epidemiology, Geisel School of Medicine, Dartmouth College, Hanover, NH 03755 USA; 2 Department of Molecular and Systems Biology, Geisel School of Medicine, Dartmouth College, Hanover, NH 03755 USA; 3 Department of Epidemiology, Brown University, Providence, RI 02912 USA; 4 Department of Pathology and Laboratory Medicine, Brown University, Providence, RI 02912 USA

## Abstract

A new mitotic clock and mathematical approach that incorporates DNA methylation biology common among human cell types provides a new tool for cancer epigenetics research.

## Introduction

The ability to accurately and efficiently detect the acceleration in the stem cell mitotic “tick rate”—a so-called mitotic clock—could be a useful tool for predicting cancer risk. It has long been recognized that mitosis itself is highly mutagenic [1, 2] and enhanced mitosis in a stem cell may be an important factor in cancer risk. To date, mitotic clock models that use genetic approaches such as telomere length have not consistently predicted risk, potentially in part because of differences in functional programming among cell types. Previous epigenetic mitotic clock models included ones that used random replication errors at CpG sites as signals of mitotic activity, allowing stem cells to be followed retrospectively [3]. Although these epigenetic mitotic clocks were ingenious in design, their practical application in human tissues has been limited by the need to sample stem cells from multiple tissues directly. Hence, while it is clear that a quantitative estimate of mitotic activity in stem cells is likely to be strongly associated with cancer risk, knowledge of biomarkers specific to stem cells that signal mitotic activity has been lacking. A new, biologically based approach presented in the current issue of *Genome Biology* [4], called epiTOC, uses an integrative methodology that makes use of previous work on estimation of tissue-specific stem cell division rates and devises a model for an epigenetic mitotic clock that overcomes these challenges.

In formulating the new epiTOC tool, Yang and colleagues [4] take on the task of identifying putative phenotypically important variation in DNA methylation that is related to both stem cell alterations and disease risk. It can be extremely challenging to differentiate cell- or tissue type-specific events that are associated with disease risk from effects that are common across cell types because differences in patterns of DNA methylation among normal cells and tissue types are incompletely characterized. Epigenetic events that occur at loci related to “stemness”, lineage-specific differentiation events, or cell-specific responses to transcription factors can depend on cell or tissue type, whereas events that occur at loci associated with metabolic and genetic regulation may be shared among cell types. At the same time, much progress has been made in the search for epigenetically important cancer disease risk loci. While early candidate gene studies comparing tumors with normal cells identified gene-specific hypermethylation (primarily in promoter regions) and DNA nucleotide repeat element hypomethylation (genome-wide hypomethylation), recent high-resolution approaches [5] have shown promise for assessing epigenetic variation in multiple normal and abnormal cells and tissues. Subsequent work from experimental studies has given us better maps relating the genomic context of CpG DNA methylation to functional gene regulation. This sets the stage for accelerated development and testing of potentially useful non-genetic, DNA-based biomarker tools in healthy and diseased cells. More specifically, we are becoming better positioned to recognize signals that are informative for specific types of questions. The integration of cell type data and an epigenetic approach to “telling time” has improved the coordinated universal model of keeping mitotic time by adding guidelines for adjusting to the right “time zone”. Indeed, here Yang and colleagues [4] apply knowledge of the stem cell functional phenotype of polycomb-related genes and integrate this with variation over calendar time to discover loci that are putatively related to mitosis.

## The epigenetic clock as a tool for cancer risk prediction

In this work, Yang and colleagues [4] select specific Polycomb target loci that are both unmethylated in multiple fetal tissues and show age-associated hypermethylation and hypothesize that methylation at these sites reflects relative mitotic activity. They then construct a model that shows that cancer and pre-cancer tissues have increased DNA methylation relative to relevant normal tissues. This, they posit, reflects enhanced stem cell activity and increased cancer risk.

## Modeling assumptions are limited by current knowledge

Yang and colleagues [4] are to be congratulated for combining cutting-edge biological knowledge with state-of-the-art bioinformatics in building a cancer prediction model. Scrutiny of this provocative model is certain to result in modifications and refinements to it as the underlying assumptions (of both the model and past experiments) are challenged and the understanding of the underlying biology improves. At the outset, we note that there are a few important assumptions and limitations in this work.

First, the stem cell division rates applied in this work are derived from those presented in Tomasetti and Vogelstein [6]. While this is reasonable, as Tomasetti and Vogelstein indicate in their work [6] there is room for improvement in the estimates they present. In addition, the current model is tested in cancer tissues and shows universal increases, but the authors have not yet shown evidence of prediction of risk in a prospective setting, where availability of data is still very limited. The current model does not include or apply estimates of the contribution that somatic alterations in non-stem cells (in any tissue or tissue environment) may make to tick rate. Altered somatic cells, particularly in tissues with higher levels of carcinogen exposure, may have non-stem cells that propagate alterations and increase the estimated tick rate of the mitotic clock. We also note here that the potential contribution of immunity and inflammation, which are particularly important in many solid tumors, is not yet specifically included in the model. The current model is also built, appropriately, using data from just one tissue source, and additional methylation data from normal tissues in healthy subjects are needed to expand and further examine the predictions of the model. Finally, reference-free and reference-based approaches would have to be properly applied to adjust for cellular heterogeneity in the setting of various other normal tissue types.

We also highlight that, as the authors note, this model necessarily assumes that methylation at the informative loci occurs only in stem cells. This is novel biology for which there is little to no experimental evidence. If true, it would imply that locus specificity in methylation is differentially determined in numerous distinct cellular and tissue-specific compartments.

## Implications of epiTOC

An important aspect of the work presented by Yang and colleagues [4] is that it highlights the differences between genome-wide association studies (GWAS) and epigenome-wide association studies (EWAS). In their work on this issue, the authors define additional building blocks of the DNA methylome, adding loci that putatively act in coordination and display a novel “mitotic clock” phenotype. These loci, as well as others that have been previously defined (e.g., the Horvath “aging” loci), represent the beginning of our ability to organize the methylome into distinct loci-driven phenotypic units [7]. Importantly, future discovery-based interrogation seeking to define the nature of differences in tissues or of tissues within population groups can now begin to group loci for testing rather than treating them independently. These groups can be compared for differences in the locus-associated phenotype, offering potential for better interpretation of some of the results of these studies. This is not unlike applying the now-standard techniques for delineating cell types within tissues [8–10], which represented the initial attempts to arrange epigenetic data into organizational, phenotypically defined units.

Adding these new building blocks allows us to imagine new approaches to future studies, including examination of the dynamic changes to the epigenome over the life course. For example, does epiTOC provide a window into variation in the extent to which chronological age contributes to cancer risk? The ability to estimate mitotic tick-rate acceleration has value for researchers collecting DNA methylation data, as it provides a directed approach to investigating age-correlated cancer risk and may inform on the biology of cancer risk factors studied across the life course.

## Conclusions

Tests of epiTOC’s association with increased cancer risk require case control approaches or, ideally, prospective studies. Initially, such work will be limited to cell types from the type of biological specimens that are more routinely collected in these studies, such as peripheral blood, and as epiTOC was developed using peripheral blood as the model tissue it may show initial success in that setting. In the future, other relatively accessible biospecimens could be collected in medical and research settings with a prospective design, so that cancer risk for the relevant organ could be tested. These tissues may include epithelial cells from colorectal tissue, the bladder, sputum, the cervix, and the oral cavity or ductal epithelial cells from the breast. Thus, epiTOC is a new tool of the best kind—one that immediately brings new questions to the fore while also providing the potential means to find answers to pressing old ones. It is an excellent demonstration of the power of incorporating biology into a computationally sophisticated analytic framework.

## Abbreviations

epiTOC: Epigenetic timer of cancer
